# A three-dimensional quantitative assessment on bony growth and symmetrical recovery of mandible after decompression for unicystic ameloblastoma

**DOI:** 10.1038/s41598-024-66411-4

**Published:** 2024-07-05

**Authors:** Tingwei Bao, Di Yu, Jiaqi Zheng, Wenyuan Zhu, Dong Wei, Huiming Wang

**Affiliations:** 1 Department of Oral and Maxillofacial Surgery, The First Affiliated Hospital, Zhejiang University School of Medicine, #79 Qingchun Road, Hangzhou, Zhejiang China; 2 General Department, Hangzhou Dental Hospital, # 1 Pinghai Road, Hangzhou, Zhejiang China; 3 The Affiliated Hospital of Stomatology, School of Stomatology, Zhejiang University, #395 Yan’an St, Hangzhou, 310000 Zhejiang China

**Keywords:** Decompression, Ameloblastoma, Volume reduction, Bone amount, Cortical perforation, Symmetry, Oral diseases, Medical imaging

## Abstract

Unicystic ameloblastoma (UAM) of the jaw can be effectively reduced in volume through decompression, which promotes bone regeneration and restores jaw symmetry. This study quantitatively evaluated changes in mandible volume and symmetry following decompression of mandibular UAM. This study included 17 patients who underwent surgical decompression followed by second-stage curettage for mandibular UAM. Preoperative and postoperative three-dimensional computed tomography (CT) images were collected. Bone volume and the area of cortical perforation were measured to assess bone growth during decompression. Mandibular volumetric symmetry was analyzed by calculating the volumetric ratio of the two sides of the mandible. Twelve pairs of landmarks were identified on the surface of the lesion regions, and their coordinates were used to calculate the mean asymmetry index (AI) of the mandible. Paired *t*-tests and the Mann–Whitney *U* test were used for statistical analysis, with p < 0.05 considered indicative of statistical significance. The mean duration of decompression was 9.41 ± 3.28 months. The mean bone volume increased by 8.07 ± 2.41%, and cortical perforation recovery was 71.97 ± 14.99%. The volumetric symmetry of the mandible improved significantly (p < 0.05), and a statistically significant decrease in AI was observed (p < 0.05). In conclusion, UAM decompression enhances bone growth and symmetry recovery of the mandible. The present evaluation technique is clinically useful for quantitatively assessing mandibular asymmetry.

## Introduction

Ameloblastoma is a benign but locally infiltrative odontogenic tumor accounting for approximately 9–11% of all odontogenic tumors^1–3^. Its growth can lead to bone destruction and mandibular deformities. Cystic ameloblastoma comprises about 5–22% of all ameloblastoma variants^4,5^. While invasive surgical treatments, such as bone resection, are commonly considered the treatment of choice, they can result in serious complications, including facial deformities, maxillary bone fractures, and dental losses. There is a general consensus that cystic ameloblastoma can be treated conservatively to avoid jaw resection^6–9^.

Both marsupialization and decompression are effective conservative treatments for large unicystic ameloblastomas (UAMs)^7,10^. These treatments aim to decrease the size of extensive cystic lesions prior to curettage by reducing intracystic pressure, thereby making second-stage surgery less invasive and safer. The main advantages include avoiding pathological fractures and bone resection, reducing intraoperative trauma and bleeding, and preserving adjacent structures such as the mandibular alveolar nerve and teeth^1,11^. Unlike marsupialization, decompression uses devices to keep the cystic cavity open, which can accelerate lesion reduction through active decompression^12,13^.

During decompression, primary changes include shrinkage of the lesion and the formation of new bone. The therapeutic effect of decompression is mainly assessed using radiographic imaging, such as CT and panoramic imaging^6,14^. Virtual measurement is commonly used to calculate the volumes of lesions^3,4,14,15^. As treatment progresses, new bone forms in the cavity, promoting changes in mandibular symmetry and contour. However, quantitative evaluation of these changes has rarely been reported^16^. Accurately measuring the post-decompression recovery of mandibular symmetry is challenging due to the irregular cortical defects and complex three-dimensional (3D) contour of the mandible.

In the present study, we developed a quantitative method to evaluate the bony volume and symmetry of the mandible following UAM decompression and to verify its efficacy by assessing postoperative improvement.

## Materials and methods

This retrospective study included 17 patients with histologically diagnosed UAMs of the mandible, treated between 2016 and 2023 at the Department of Oral and Maxillofacial Surgery, First Affiliated Hospital, Zhejiang University School of Medicine. The study protocol was approved by the Clinical Research Ethics Committee of the First Affiliated Hospital, Zhejiang University School of Medicine. Informed consent was obtained from patients and/or their legal guardians. This cohort study was conducted in accordance with the ethical standards of the responsible committee on human experimentation and the Declaration of Helsinki.

This study included patients with initial lesions having a major diameter of > 4 cm located in the unilateral posterior mandible or ramus, as confirmed by X-ray or CT, and a pathological diagnosis of UAM. Patients were excluded if they had a recurrent ameloblastoma, acute infection, pathological fracture, or multilocular lesions. The basic information of the patients is presented in Table 1.

**Table 1:** Demographic and clinical characteristics of patients. Data are presented as mean ± SD or number of patients.

Variable	Value
Gender (M:F)	8:9
Age (years)	19.82 ± 8.1
Duration (months)	9.53 ± 3.22
Initial lesion volume (cm^3^)	24.02 ± 17.11
Initial cortical perforation volume (cm^2^)	31.66 ± 15.37
Initial bone volume (cm^3^)	63.42 ± 11.44
Extraction of involved teeth (yes:no)	12:5
Root resorption (yes:no)	12:5

### Surgical procedure

The treatment protocol involved initial decompression followed by secondary curettage of the UAM.

**Figure 1:**
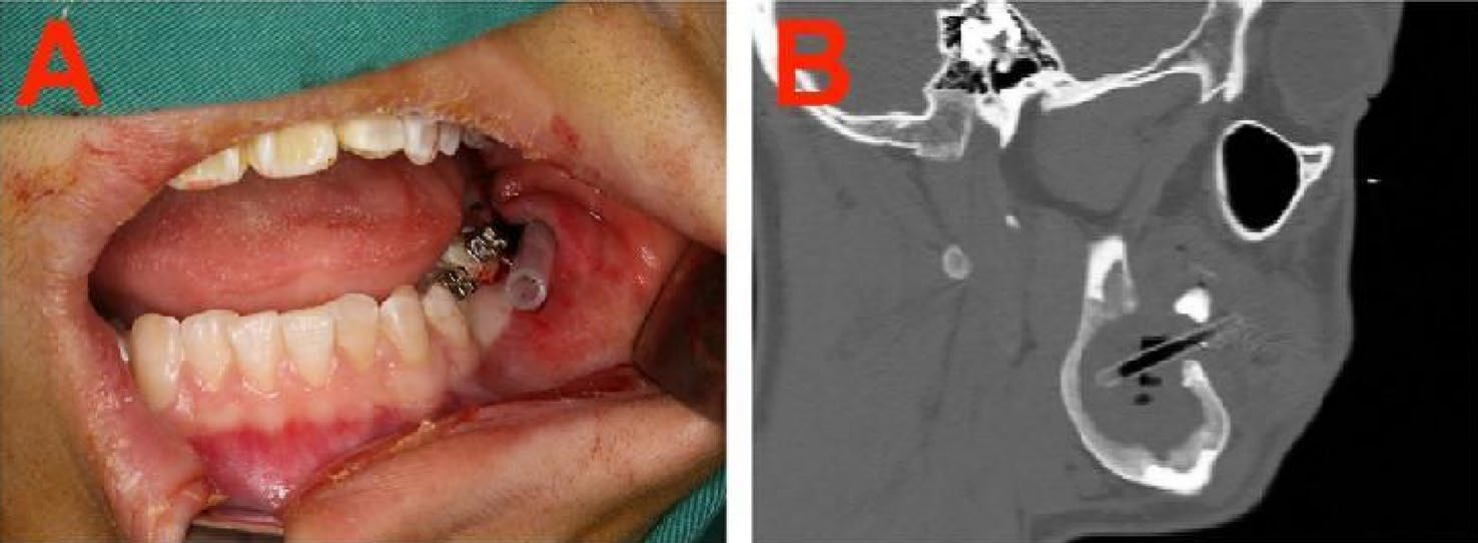
Decompression was maintained using a silicone tube. (**A**) Intraoral view. The decompression tube was fixed to the bracket on an adjacent tooth using a stainless steel ligature wire. (**B**) CT view. The decompression tube was inserted into the deep side of the lesion cavity through the opening.

Under local anesthesia, decompression was performed through intraoral incisions in the region of swelling or alveolar crests. A portion of the overlying bone, mucoperiosteum, and cystic wall was excised to release intracystic pressure, and the specimen was sent for biopsy. A 4-mm-wide silicone tube was inserted into the lesion through the opening to maintain decompression (Fig. 1A). The other end of the tube remained in the oral cavity and was anchored to a bracket on an adjacent tooth using a stainless steel ligature wire (0.1000 Krugg ligature). Patients were instructed to irrigate the lesion cavities twice a day through the tube with normal saline using 20-mL syringes.

After the decompression surgery, patients were instructed to present for monthly follow-up visits. During these visits, the surgeon checked the patency of the tube and assessed the tumor size. Once the tumor had reduced in size and excess drainage was observed, the tube was replaced with a shorter one if necessary. A 64-slice CT of the mandible was performed every 3 months (Fig. 1B). Decompression continued until sufficient bone had formed in the cavity, as evidenced by new bone overlying the inferior alveolar nerve and sufficient bony thickness to prevent pathological fractures (i.e., at least 1.5 cm from the bottom of the mandible).

Second-stage curettage of the remnant tumor was performed under general anesthesia. Any impacted teeth involved in the lesion were also extracted. Additionally, approximately 0.3–0.5 cm of bone surrounding the tumor was removed to reduce the recurrence rate.

After the second-stage curettage, follow-up was performed every 3 months in the first year, every 6 months in the second year, and annually thereafter.

## Outcome evaluation

**Figure 2:**
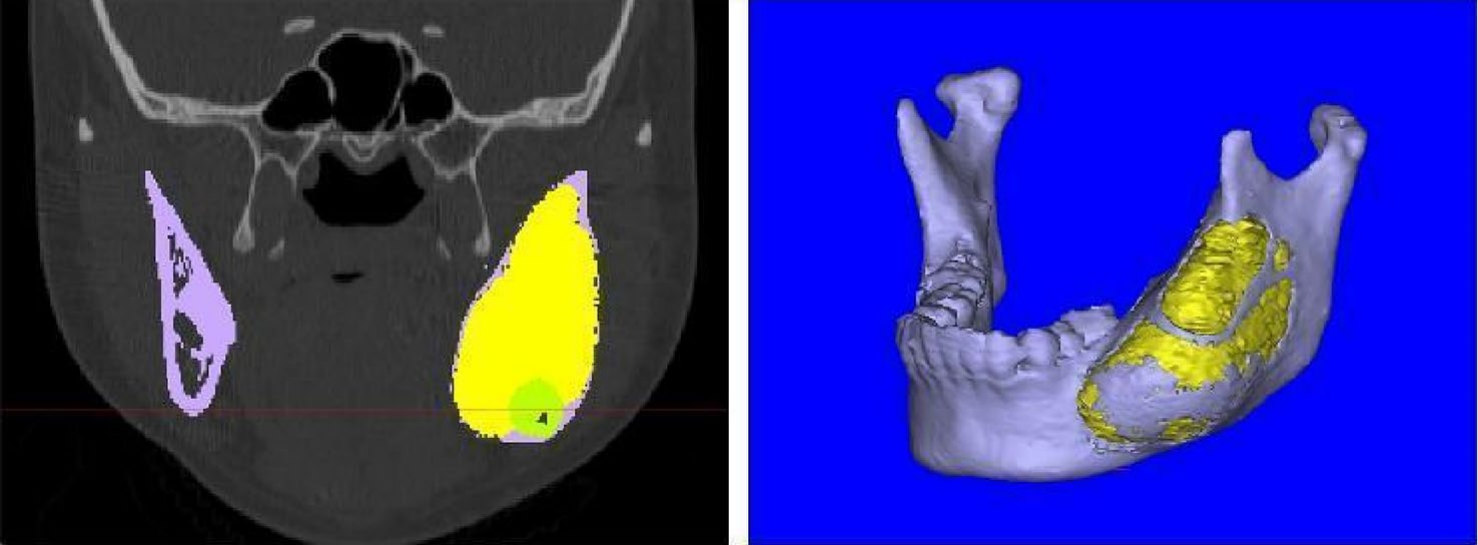
Reconstruction of a 3D model of the mandible, impacted tooth, and lesion. (**A**) Coronal CT images of the mandible (purple), lesion (yellow), and impacted tooth (green) were selected. (**B**) A 3D image of the mandible showed severe swelling and resorption of the mandibular ramus and body, with a large defect in the cortical layer.

CT data (CT 64 Slice; Philips Medical Systems, Best, the Netherlands) were collected before and after decompression. The scanning parameters were as follows: 0.75 mm slice thickness, 100 mAs, 120 kVp, and a 512 × 512 image matrix size. CT images were stored in Digital Imaging and Communications in Medicine format and subsequently imported into Mimics 13.1 (Materialise NV, Leuven, Belgium) to construct virtual 3D models of the tumors, impacted teeth, and mandibles (Fig. 2). The 3D models were rendered by summing each CT slice and then exported in STL format. The STL files were imported into 3-Matic (Materialise NV) for quantitative analysis. The volume (mL) of the tumor or mandible was measured automatically by the software.

### Bony volume measurement

The bony volumes of mandibles were measured excluding the tumors and impacted teeth. Postoperative change in bone volume (mL) was calculated as final bone volume − initial bone volume. Postoperative relative change in bone volume (mL) was calculated as (final bone volume − initial bone volume) × 100%/initial bone volume.

### Area of cortical perforation

**Figure 3:**
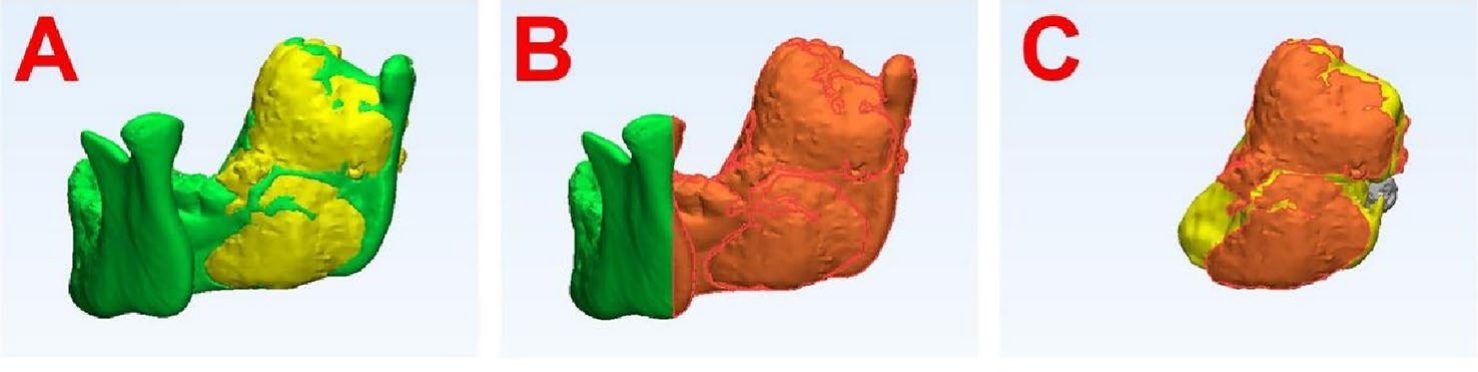
Measurements of the area of cortical perforation in a representative case. (**A**) Alignment of the mandible and lesion. (**B**) Markings of the cortical layer, including the perforation. (**C**) The cortical perforation was projected to the lesion surface for measurement.

The region of cortical perforation was marked and projected to the lesion surface (Fig. 3).

Subsequently, the area of cortical perforation (cm^2^) was calculated as the initial area of cortical defect − the final area of cortical defect. 

Furthermore, the relative reduction in the area of the cortical perforation (cm^2^) was calculated as the (initial area of cortical perforation − final area of cortical perforation) × 100%/initial area of cortical perforation.

### Volumetric analysis

The reconstructed mandible model was divided into two hemi-mandibles along the facial sagittal plane: lesion and healthy area. Each part was assessed for average volumetric discrepancy. Volumetric symmetry was calculated as the lesion health volumetric ratio, indicating the degree of similarity between the two parts:

$$ \begin{aligned} &{\text{Volumetric ratio}} = {\text{bone volume of the lesion}} \times 100\% {\text{/bone volume of the healthy part}}. \\ &{\text{Volumetric discrepancy}} = {\text{bone volume of the healthy part }} - {\text{ bone volume of the lesion}}. \hfill \\ \end{aligned} $$

### Landmark-based mandibular symmetry analysis

Prior to analysis, a standard symmetrical mandible was constructed by mirroring the healthy part of the mandible and the lesion part. The median sagittal plane was considered the reference plane.

**Figure 4:**
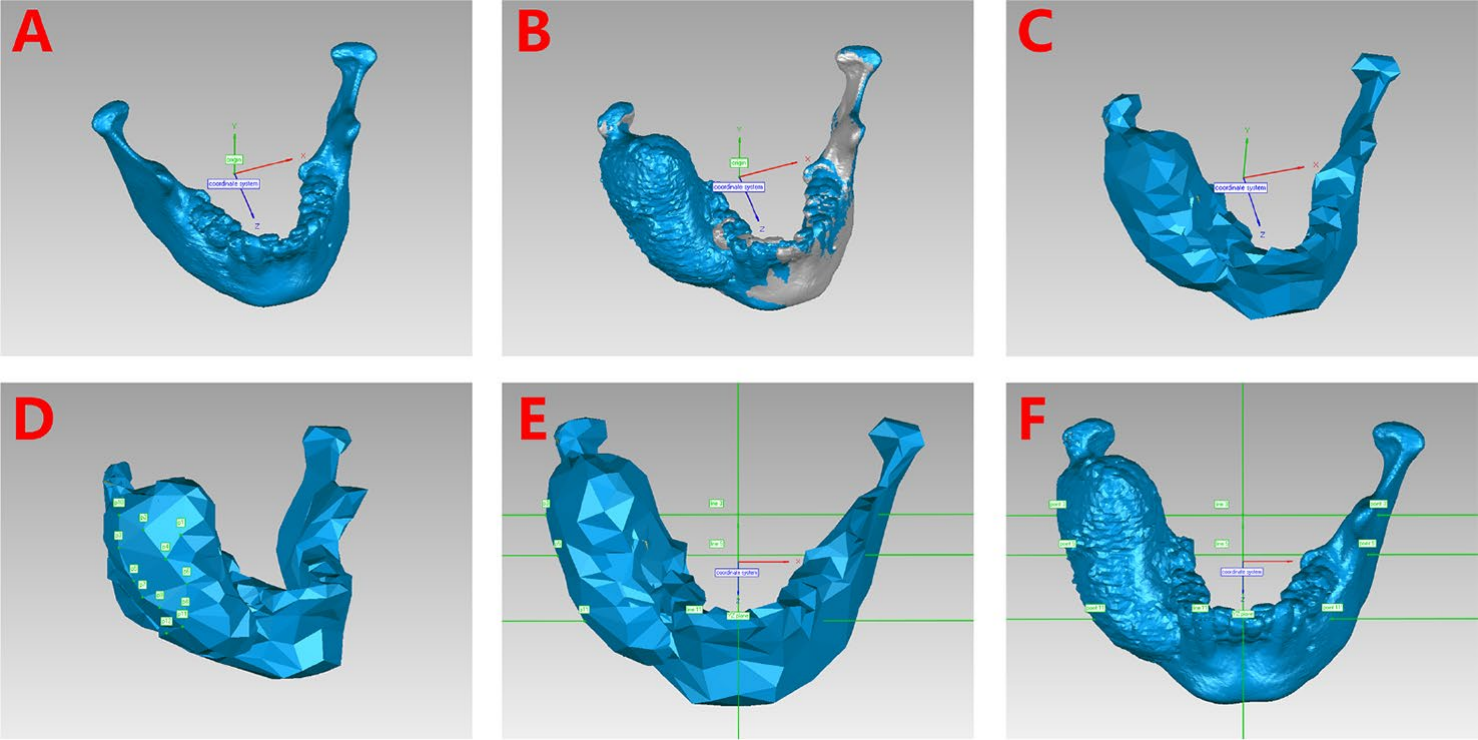
Identification of landmarks for AI analysis. (**A**) Fabrication of the 3D coordinate system. (**B**) Alignment of the pre- and post-decompression mandible and the standard symmetrical mandible. (**C**) The preoperative mandible was remeshed. (**D**) The measurement landmarks were marked using nodes on the remeshed model. (**E**) Lines passing through the landmarks perpendicular to the YZ plane were marked. (**F**) Corresponding positions of landmarks on real mandibles.

A 3D coordinate system was constructed based on the standard symmetrical mandible using Geomagic Studio (version 14.0; Geomagic, Morrisville, NC, USA) (Fig. 4A). The median sagittal plane was designated the YZ plane. The *x-*axis was defined as the line connecting bilateral gonions. The origin of coordinates (0, 0, 0) was the intersection between the *x-*axis and the YZ plane. The *y*-axis was drawn in the YZ plane, extending from the common origin toward the condylions. The *z-*axis included the common origin and was perpendicular to the XY plane. The pre- and postoperative CT models were superimposed onto the standard symmetrical mandible by aligning the nonsurgical parts (Fig. 4B).

The preoperative mandible was remeshed (Fig. 4C). Twelve landmarks were designated using the nodes on the remeshed model, ensuring a uniform distribution of landmarks on the lesion area (Fig. 4D). Their corresponding positions on different mandibular models were identified by extending a line perpendicular to the YZ plane and intersecting the surface of the aligned mandibles (Fig. 4E). Similarly, the contralateral positions of the landmarks were determined by the intersections of the perpendicular lines and the bone surface on the healthy part (Fig. 4F). Therefore, 10 pairs of landmarks were generated, with each pair ideally distributed symmetrically.

The X, Y, and Z coordinates of the landmarks were recorded. Similar to Huang et al.^17^, the asymmetry index (AI) was calculated as follows: $\sqrt{{\left(Xl+Xr\right)}^{2}+{\left(Yl-Yr\right)}^{2}+{\left(Zl-Zr\right)}^{2}}$, where X, Y, and Z are the coordinates of a landmark, *l* indicates the left side, and *r* indicates the right side. A pair of perfectly symmetrical landmarks would have an AI of 0. Lower AI values indicate higher degrees of symmetry. The average AI for the landmarks was calculated at different time points.

Two examiners independently performed landmark identification and Al measurements following the same protocol. Both examiners were trained to use the software prior to the study.

### Statistical analysis

Data are presented as mean ± standard deviation (SD). Statistical analysis was conducted using SPSS software (version 19.0; IBM Corp., Armonk, NY, USA). Paired *t*-tests were employed when data were normally distributed, whereas the Mann–Whitney *U* test was used when landmark-based AIs were not normally distributed. P-values < 0.05 were considered indicative of statistical significance.

The inter-examiner agreement of AI measurements was assessed using the intraclass correlation coefficient. Based on the 95% confidence interval (CI) of the estimated intraclass correlation coefficient (ICC), the following classifications were devised: 0.00–0.49 = poor agreement, 0.50–0.74 = moderate agreement, 0.75–0.89 = good agreement, and 0.90–1.00 = excellent agreement.

## Results

The mean duration of decompression was 9.53 ± 3.22 (range: 3–15) months. All patients experienced visible growth of new bone and a decrease in tumor size during decompression, leading to improved facial symmetry. The mean follow-up duration after the secondary surgery was 19.34 (range: 14–41) months. Recurrence occurred in one patient at 10 months after curettage, resulting in a recurrence rate of 5.88%. The patient underwent repeat curettage for the recurrent tumor and has remained free of recurrence for 33 months to date.

As presented in Table 2, the lesion volume significantly decreased during decompression (p < 0.05). Conversely, the mandibular volume was significantly increased (p < 0.05), with a relative change in bone volume of 8.07 ± 2.41%. Additionally, the area of cortical perforation decreased significantly (p < 0.05), with a relative reduction of 71.97 ± 14.99%.

**Table 2:** Changes in bone volume, area of cortical perforation, and lesion volume.

	Pre-decompression	Post-decompression	p value
Bone volume (mL)	63.42 ± 11.44	69.35 ± 12.07	< 0.05
Cortical perforation (cm^2^)	31.66 ± 15.37	8.05 ± 4.48	< 0.05
Lesion volume (mL)	24.02 ± 17.11	9.33 ± 8.86	< 0.05

The changes in symmetry are summarized in Table 3. The volumetric discrepancy of bilateral mandibular bone volume was 5.77 ± 2.75 mL. Following decompression, this discrepancy decreased significantly to 3.13 ± 2.01 mL (p < 0.05). Initially, the volumetric ratio was 83.96 ± 8.03%. After decompression, the volumetric ratio increased to 98.27 ± 10.98%.

**Table 3:** Symmetry analysis of the mandible before and after decompression.

	Pre-decompression	Post-decompression	p value
AI	13.99 ± 5.51	6.92 ± 4.79	0.001
Volume discrepancy (mL)	5.19 ± 2.92	2.59 ± 2.22	< 0.05
Volume ratio	84.68 ± 8.02%	102.14 ± 10.08%	< 0.05

After decompression, a significant reduction in AI was achieved (p < 0.05). The intra-examiner reliability of AI measurements was excellent (0.90–0.99), with an ICC of 0.926 before decompression and 0.938 after decompression.

## Discussion

Ameloblastoma predominantly affects young to middle-aged individuals, necessitating minimally invasive methods with low morbidity and long-term disease-free survival^18^. Marsupialization or decompression has been employed for this purpose, as these techniques are associated with a reduced risk of damaging bone structures. Therefore, quantitative analysis of bone remodeling using these procedures may facilitate treatment planning and provide evidence-based recommendations for managing cystic ameloblastomas. In the present retrospective study, we analyzed the effects of decompression on bony changes, revealing that decompression significantly stimulates bone growth and improves mandibular symmetry.

As decompression progresses, fundamental changes include shrinkage of lesions and the growth of new bone. The amount of newly formed bone in the cavity directly influences the risk of injuries necessitating secondary surgeries, such as pathological fractures and nerve injuries. Second-stage curettage can only be safely performed when sufficient bone has formed. Demirsoy et al.^19^ and Zhao et al.^20^ used grayscale values of panoramic images to visualize new bone formation. However, such measurements of bone volume were indirect, making it difficult to precisely assess the volume of new bone. In the present study, discrepancies between two mandibular volumes were used to estimate the volume of newly grown bone. The results showed that bone volume continuously increased throughout the decompression procedure. These changes contributed to the recovery of mechanical strength in the lesion area.

Cortical perforation is an important radiological feature in the management of ameloblastoma^18,21^. Lesions associated with cortical perforation may indicate a potential risk of postoperative recurrence^22^. One possible reason for this is that the barrier function of the cortical bone with respect to the lesion disappears once the cortical layer is damaged, allowing the ameloblastoma to adhere to the periosteum. The recovery of cortical perforation confines the boundary of the tumor, ensuring that secondary curettage of the residual tumor is precise and clear. However, precise measurement of cortical perforation is rarely reported because of difficulties in accurately identifying the complex contours of defects. In this study, the cortical defect area was projected onto the surface of the lesion, simplifying the measurement process.

Several different methods exist for measuring facial asymmetry, but a standard method has not yet been established^23,24^. Volume discrepancy has been shown to be a sensitive method for assessing mandibular asymmetry^25,26^. In this study, volume discrepancy significantly decreased during decompression, indicating a reduction in bony differences between the healthy and affected halves of the mandible. Additionally, the volumetric ratio between the two sides of the mandible was calculated. Interestingly, the ideal volumetric ratio was expected to be 100% in a perfectly symmetrical mandible, but this value was exceeded after decompression in this study. Thus, we believe that volume-based analysis primarily reflects the similarity of the bilateral bone amount but may not fully represent the symmetry of the mandibular contour.

The AI is another measure of the extent of symmetry in both bones and soft tissues^24^. Unlike volume analysis, the AI directly reflects the degree of symmetry of the mandibular contour. Common methods often rely on the Euclidean distance matrix analysis of anatomical landmarks that influence the facial outline. For example, Cao et al.^27^ used landmark-based scoring analysis to evaluate chin asymmetry. Similar methods were also employed in our previous studies to quantitatively assess changes in zygomatic symmetry after surgeries^28^. In this study, ameloblastomas occurred in different sites of the mandible, making it difficult to find suitable anatomical landmarks in all cases. To reduce bias, the lesion area was remeshed, and 12 nodes were uniformly distributed to identify landmarks. The mandible and lesion models were merged to facilitate application of the AI to the cortical defect region. The results showed that the degree of symmetry of the mandibular surface in the lesion area increased after decompression. ICC analysis demonstrated high reproducibility of this technique.

Decompression of ameloblastoma entails several risks. First, it has a higher recurrence rate compared to radical treatment^27,29,30^. However, secondary surgery in cases of recurrent cystic ameloblastoma has proven effective^30^. Nevertheless, the recurrence rate should not be the sole consideration given the benign nature of ameloblastoma. Radical surgery can result in facial deformity and dysfunction, negatively impacting facial growth, which should also be considered before treatment^31^. Second, accelerated growth of the solid component of ameloblastoma may unpredictably occur after long-term decompression or marsupialization. Yang et al.^32^ described five cases of such occurrences during marsupialization ultimately requiring wide resections. Therefore, it is crucial to closely monitor changes in lesions and bones during decompression, as well as consider the need for radical treatment if the tumor becomes enlarged.

The major limitation of this study was its retrospective nature, such that selection bias could not be avoided. Additionally, this study had a small sample size. Considering that our findings provide only preliminary evidence, further prospective trials using larger samples are needed.

This study describes 3D quantitative analysis for evaluating mandibular volume and symmetry, offering significant practical value for clinical use. The combination of volumetric analysis and landmark-based AI calculation facilitates the quantification of symmetry in cases with complex anatomy. In the future, this technique can be further utilized in research and enhanced through the integration of artificial intelligence. In conclusion, decompression for UAM can significantly benefit bone recovery and improve mandibular symmetry. Special attention should be paid to effectiveness and recurrence throughout the entire decompression process and postoperative follow-up.

## Data Availability

The data used and/or analyzed during this study are available from the corresponding author on reasonable request.
